# Dung beetle community composition affects dung turnover in subtropical US grasslands

**DOI:** 10.1002/ece3.8660

**Published:** 2022-02-22

**Authors:** Roisin Stanbrook, Joshua R. King

**Affiliations:** ^1^ 6243 Biology Department University of Central Florida Orlando Florida USA

**Keywords:** agroecosystems, dung beetles, dung degradation, subtropical grasslands

## Abstract

1. An important service in many ecosystems is the turnover and degradation of dung deposited by cattle. Dung beetles are the primary group of insects responsible for dung turnover, and factors affecting their abundance and distribution thus impact dung degradation. Lands lost to grazing due to dung buildup and pasture contamination total millions of acres per year in US pastures.
2. We evaluated the structural differences in dung beetle assemblages in natural grasslands versus a managed agroecosystem in subtropical southeastern Florida (USA). We measured the direct effect of dung longevity when dung beetle fauna normally inhabiting dung pats were excluded.
3. Our results indicate dung beetle abundance, functional diversity, and species richness have a substantial impact on the rate of dung turnover in subtropical pastoral lands with ~70% of dung removed from the soil surface after three months. Functional diversity and evenness did not have a significant positive effect on dung removal in managed, versus natural grasslands demonstrating a strong relationship between dung beetle assemblage composition and delivery of a key ecological process, dung degradation.
4. We suggest the importance of trees, which provide a thermal refuge for beetles, should be dispersed within matrixes of open pasture areas and within proximity to adjacent closed‐canopy hammocks to facilitate the exchange of dung beetles between habitats and therefore maintain the provisioning of dung degradation services by dung beetle assemblages.

## Graphical abstract

An important service in many ecosystems is the turnover and degradation of dung deposited by cattle. Dung beetles are the primary group of insects responsible for dung turnover, and factors affecting their abundance and distribution thus impact dung degradation. Our results indicate dung beetle abundance, functional diversity, and species richness has a substantial impact on the rate of dung turnover in subtropical pastoral lands with ~70% of dung removed from the soil surface after three months.

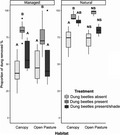

## 1 INTRODUCTION

Ecosystem functions may be defined as the capacity of natural processes and components to provide goods and services that satisfy human needs, either directly or indirectly (de Groot et al., 2002). Ecosystem functions deemed important to for sustaining humanity are termed “ecosystem services.” Insects impact a broad variety of ecosystem functions and thus their activities are crucial in the provision of several ecosystem services (Beynon et al., 2012). Such services may include benefits for primary production, pollination, seed dispersal, biological pest control, nutrient cycling, and water quality. Insects play key roles in the regulation and dynamics of many ecosystem services but the extent and actual quantity of their impact is often presumed with little experimental verification (Noriega et al., 2018). An important service in many ecosystems is the turnover and degradation of dung deposited by large herbivores, and especially cattle, in a variety of natural grassland and managed agroecosystems, worldwide. Approximately 70%–90% of the nitrogen (N), phosphorus (P), and potassium (K) ingested as feed by forage grazing cattle can be recovered in excreted dung and urine (Haynes & Williams, 1993; Williams & Haynes, 1990). Understanding the primary controls on the movement of dung into the soil from the ground surface thus has important implications for nutrient cycling and overall ecosystem function. This is especially true for natural and managed grassland agroecosystem ecosystems, worldwide, where beef and dairy production are dependent upon the provision of key services, like dung turnover.

Dung beetles (Coleoptera: Scarabaeinae) are the organisms primarily responsible for the acceleration of dung removal from ground surfaces and are thus essential for maintaining key ecosystem functions in grassland agroecosystems (Braga et al., 2013; Louzada & Silva, 2009). They bury the dung of mammals for both nesting and feeding purposes (Hanski & Cambefort, 1991; Stanbrook, 2020), an important ecological function that is easily understood in the context of ecosystem service provisioning. Their activities directly affect nutrient cycling (Nichols et al., 2008; Stanbrook et al., 2021; Yamada et al., 2007), improvements in soil fertility and physical characteristics (Hea et al., 2005), fly and gastrointestinal parasite reduction (Braga et al., 2012; Nichols & Gómez, 2014), increases in plant growth and recruitment (Badenhorst et al., 2018), reductions of greenhouse gas emissions (Penttilä et al., 2013), and seed dispersal (Vulinec, 2000). In addition, dung beetles are also considered efficient indicators of environmental changes (Bicknell et al., 2014; França et al., 2016), often being used as focal organisms to assess anthropic and natural impacts (Costa et al., 2017; Korasaki et al., 2013).

Three main dung beetle “functional groups” have been described according to their feeding and/or nesting behavior: endocoprids (dwellers), paracoprids (tunnellers), and telecoprids (rollers) (Doube, 1990). These three different groups are ecologically functional because they describe how different groups of beetles handle and consume dung which determines where dung is relocated after deposition. In addition to functional grouping of species based on dung relocation behavior, measurements of morphological, ecophysiological, and life‐history characteristics which may affect the biological performance of individuals (e.g., functional traits) are predictive of the effect the beetles may have on their environments. Therefore, knowledge of how beetle communities changes in trait composition can be used to infer how disturbance more broadly effects on ecosystem processes through the loss of ecosystem services dung beetles provide. Multiple lines of evidence suggest that while species richness and diversity can enhance ecosystem functioning (Hooper et al., 2005), to refine predictions and mechanistic understanding of biodiversity–ecosystem function (BEF), it has been increasingly accepted that instead of focusing on the taxonomic identity of organisms, the diversity of functional traits of species within a community should be studied (Gagic et al., 2015). In dung beetles, morphological traits which relate to nesting behavior; such as body size and front and rear tibial length can be used as functional traits to determine biodiversity–ecosystem functioning (deCastro‐Arrazola et al., 2020) as these are linked to both the efficiency in which dung can be removed from the soil surface and the quantity of dung which is removed over time.

Notable declines in insect biodiversity have heightened the need to understand how declines in insect taxa affect the provisioning of ecosystem functions under different land‐use scenarios but an important question is: Does dung beetle taxonomic and functional diversity affect dung turnover rates in grassland ecosystems under different management scenarios? While this question has mostly been addressed in grasslands found in subtropical forest regions that have been altered through logging and monoculture (e.g., Sarmiento‐Garcés & Hernández, 2021) and some tropical grasslands (Correa et al., 2020), it has yet to be addressed in many other parts of the world that are undergoing different drivers of land‐use change, such as the subtropical United States, where land‐use conversion from undisturbed prairie to improved pastures used for cattle ranching is widespread, and habitat loss through draining and damming of existing grasslands for construction and housing development is accelerating at an alarming rate (Hernández et al., 2012; Marshall et al., 2003). Additionally, the taxonomic dung beetle species found in Florida form part of a varied population of adventive and established non‐native species which were intentionally introduced into nearby states (Wood & Kaufman, 2008) and to date little research has been conducted on how these species *Digitonthophagus gazella* and *Euoniticellus intermedius* affect the rate of dung degradation in the United States (Pokhrel et al., 2021).

The most obvious evidence of lack of dung beetle activity in grazing systems is the fouling of pastures with unburied dung. Hectares lost to grazing due to dung buildup and pasture contamination total millions of lost area per year across US pasturelands. Fouling of pasture grasses by cattle dung occurs in the absence of effective processing by dung beetles. In addition to covering forage grasses, dung also may adhere to grass blades, thereby reducing palatability. The time needed for cattle dung pats to totally degrade and recycle is dependent on many factors such as season, pasture type, faunal invertebrate inhabitants, and microclimate (Lopez‐Collado et al., 2017). Total dung pat degradation can occur in as little as one month (MacDiarmid & Watkin, 1972), or take as long as 3 or 4 years in pastures when cattle have been treated with insecticides and where dung beetles populations are reduced or absent (Anderson et al., 1984). Pasture fouling through continuous dung deposition that fails to degrade quickly can represent a substantial problem to cattle ranchers if left unmanaged. Economic losses can include the need for additional provisioning with silage, and the need to use harrowing to break up and redistribute dung pats which can result in up to 18% decreases in herbage yield (Weeda, 1967). When dung pats are deposited on a pasture, a large proportion of available forage underneath and up to a 6‐m radius around the pat is unused by grazing cattle until the pat is incorporated into the soil (Fincher, 1981). This represents a substantial loss of forage available to cattle and a significant economic loss in terms of the acreage available to ranchers.

Over 11% of Florida's land cover is devoted to pasturelands (Volk et al., 2017) which support an expanding billion‐dollar beef cattle industry (Hodges et al., 2019). However, no studies have assessed the role of dung beetles in dung removal in US subtropical pastoral systems using manipulative experiments. In this study, we compared long‐term dung removal by dung beetles under field conditions in both natural and managed grasslands in the south eastern United States. We manipulated the access of dung beetles to dung pats in order to understand their importance for dung removal in managed cow‐calf operations. We compared managed pasture systems with a natural landscape containing dung beetle populations to investigate the differences in dung removal under different management conditions. Experiments were conducted to measure dung removal when: (i) dung beetles have full access to dung pats; (ii); dung beetles had full access to partially covered dung pats, and (iii) dung beetles were completely excluded from dung pats. Dung beetles were sampled from all locations to determine functional as well as species diversity. Our objectives were twofold: (1) describe the taxonomic and functional differences in dung beetle communities found in natural and anthropically managed agroecosystems, and (2) compare dung mass removal in managed versus natural grasslands under three conditions to simulate dung degradation when dung beetle fauna normally inhabiting dung pats were both present and excluded. We hypothesized that slower dung pat degradation would occur in dung pats where dung beetles were excluded and accelerated in dung pats exposed to dung beetle activity. We also expected that due to the higher functional and taxonomic diversity of dung beetles typically found in natural grassland sites, dung pats in these sites would show increased mass removal when compared to dung pats in managed pastures.

## 2 MATERIALS AND METHODS

### 2.1 Study area

To examine the effects of dung beetle taxonomic and functional diversity on dung decomposition rates, we set up a series of mesocosm experiments at three locations: two working cow‐calf operations with medium stocking densities during May–August 2019 (Durando Ranch; N27°37.310′, W80°53.268′, Buck Island Ranch; N27°817′, W81°13.676′) and at Kissimmee Prairie Preserve State Park (KPPSP) during June–August 2020 (N27°36.212′ W81°05.730′) (Figure S1). All locations are in south‐central Florida, USA. The principal natural vegetation type in the region is a mix of central Florida dry and wet prairie interspersed with improved and semi‐improved non‐native grasslands used extensively for cattle ranching. Florida dry prairies are an endemic landscape restricted to south‐central Florida (Noss, 2020). The dry prairie landscape is maintained by frequent, lightning‐season fires followed by temporary, seasonal flooding and consists of large, open expanses of prairie dominated by a diversity of grasses and forbs interspersed with low‐growing shrubs (Noss, 2013). KPPSP was previously used for cattle grazing from the 1920s until 1997 when KPPSP was established by the state of Florida (www.floridastateparks.org). However, part of the Preserve still remains grazed at a low stocking density of 1 cow‐calf pair/4 ha (Tucker et al., 2010). In the two managed grassland locations, both ranching operations seasonally manage vegetation with prescribed burns and maintain patches of hammocked vegetation within pastures to provide shade for cattle during the hot summer months. The climate is subtropical humid (Köppen climate classification) with a distinct wet season (from May to October) and dry season (from November to April).

### 2.2 Experimental design

**FIGURE 1:**
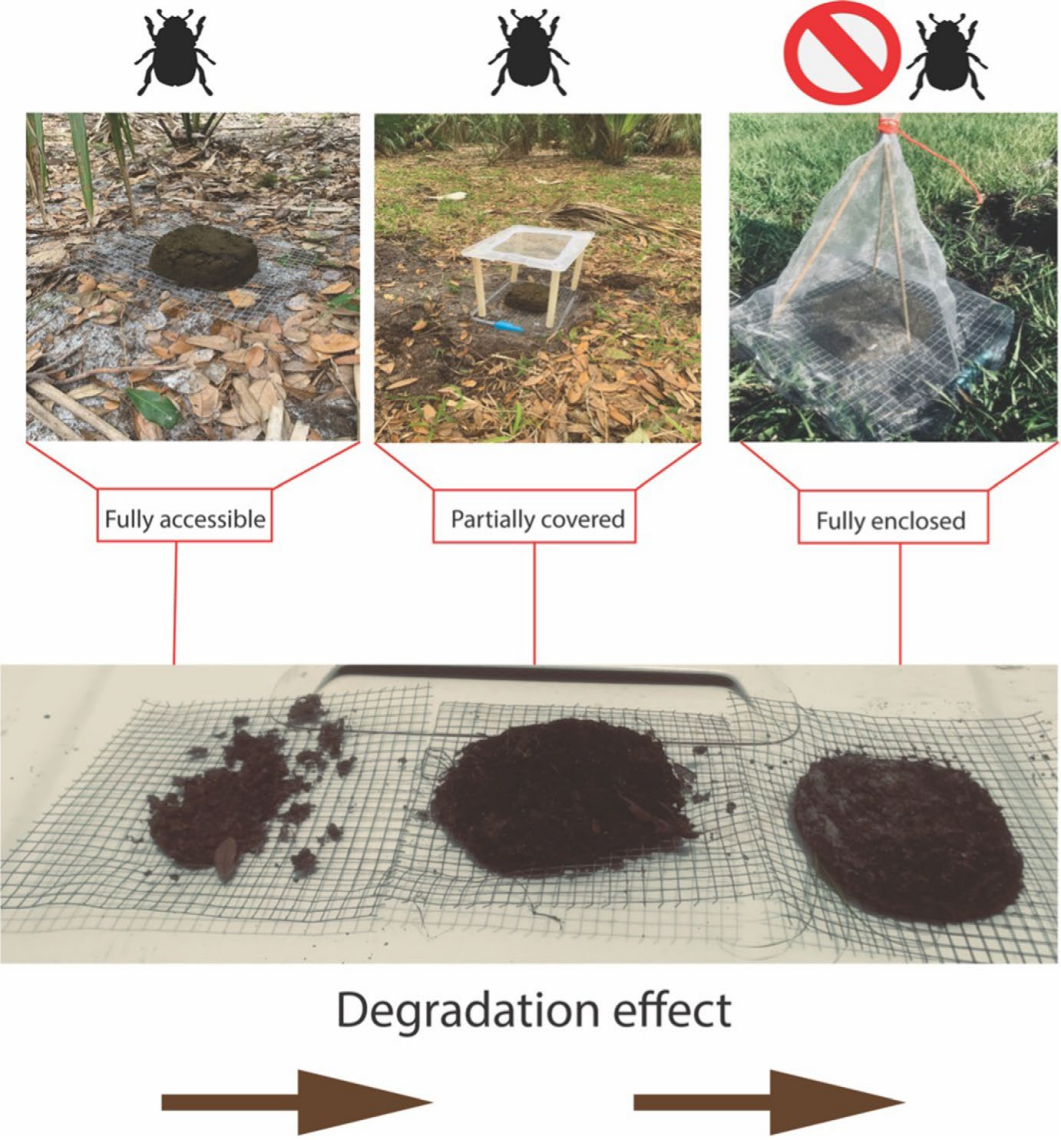
Design and typical outcomes for dung beetle access to dung treatments

Experimental plots were placed in either open or canopied hammocks in semi‐improved pasture (managed lands) and within open native dry prairie or within oak (*Quercus virginiana*)/cabbage palm (*Sabal palmetto*) hammocks in KPPSP (natural lands). Thus, the overall design consisted of 40 open and canopied plots on both natural (20 plots) and managed lands (20 plots) (Table 1). We used three treatment types within plots which consisted of dung (open access to aboveground fauna), shaded dung only (dung accessible to aboveground fauna but under a mesh cover to limit damage to dung pats by heavy rainfall), and dung inaccessible to both above and below ground fauna (Figure 1). Each treatment used 400 g defrosted and homogenized cow dung molded into a hemisphere using a 700 ml plastic container placed on a 30 × 30 cm wire grid with an aperture size of 2.5 × 2.5 cm. Plastic trays pierced with 0.33 cm drainage holes were placed beneath each experimental unit to limit any other faunal coprophilous arthropods from entering the mesocosm from below.

**TABLE 1:** Experimental design for the dung degradation experiments

Location	Number of sites		Treatment^a^	Total number of replicates
	Open Pasture	Canopy		
*Managed grassland*				
Durando Ranch	5	5	FA(10), PC(10), FE(10)	30
Buck Island Ranch	5	5	FA(10), PC(10), FE(10)	30
*Natural grassland*				
Kissimmee Prairie Preserve State Park	20	20	FA(20), PC(20), FE(20)	60

^a^ Fully accessible (FA); Partially covered (PC); Fully enclosed (FE). See Figure 1.

### 2.3 Dung beetle sampling

Dung beetles were sampled using three baited pitfall traps placed at the center of each 30 m^2^ experimental plot at the beginning and end of the study period (June and September 2019, 2020). Each trap consisted of a plastic container with a 1 L capacity (15 cm deep and 12.5 cm diameter), with the container one‐quarter filled with propylene glycol to preserve specimens. Traps were baited with 200 g cow dung and left open for four days (eight days total per plot) before being removed and the beetles enumerated and identified using the following taxonomic keys: Harpootlian (2001) and Woodruff (1973). All pitfall traps were set using standard dung beetle sampling protocols (Krell, 2007).

### 2.4 Functional diversity analyses

We used information on body size (length from clypeus to pygidium (mm), and dry body mass in (g), nesting strategy, diel activity, and microhabitat preference for functional trait analyses (Buse et al., 2018). Individual dry body mass for each species was calculated using the regression equation (body mass (mg) = 0.038 × mean body length (mm) 2.46) provided for use with terrestrial arthropods by Ganihar (1997). Overall dung beetle biomass per trap was calculated by multiplying the abundance of each species by the individual body mass estimated from the regression. Biomass of tunneller, roller, and dweller species was calculated separately. Nesting strategy was considered a combination of body size (large (>10 mm) vs. small (<10 mm)) and nesting behavior (tunneller, roller, dweller). Tunnellers and rollers relocate dung vertically and horizontally away from deposited dung pats and then the relocated dung is stored underground for feeding or in nesting chambers. Dwellers typically stay within the dung pats or within the soil directly below dung (Hanski & Cambefort, 1991). As functional diversity cannot be summarized by a single number (Mason et al., 2005), we used distanced‐based matrix of functional diversity indices (dbFD) to investigate effects of functional diversity on dung removal. dbFD returns the functional diversity indices: functional richness (FRic), functional evenness (FEve), and functional divergence (FDiv) (Villéger et al., 2008). Functional traits used for calculating dbFD were as follows: dry body mass and nesting strategy.

### 2.5 Dung removal rates

As metrics of dung removal, we took repetitive weights of dung (g) throughout the course of the experiment. Alterations in this metric are hereafter referred to as the dung removal rate. We also recorded the weight of dung (g) remaining on the surface at the end of the experiment (hereafter remaining dung mass). By using dry weight, we controlled for any difference in evaporation, thereby isolating the contribution of the insects themselves to dung removal (Rosenlew & Roslin, 2008). Importantly, dung removal rate and final remaining dung mass are complementary aspects of the removal function since one may arrive at the same final weight through steeper or shallower removal trajectories based on dung beetle species’ interaction with dung. As such, a slower removal rate results in undecomposed dung remaining on the pasture and affecting new forage growth for a longer period.

### 2.6 Data analyses

We tested relationships between dung removal (= proportion of wet weight removed from soil surface) and dung beetle species richness, overall abundance, overall beetle biomass, and functional diversity with linear mixed‐effect (LME) models (40 plots, with three observations each = 120 pats). We defined management as random effect and used habitat type; either open pasture or canopy, (“habitat effect”) and community attributes; species richness, abundance, and functional diversity (“community effect”) as main effects to control for non‐independence of individual dung pats (dung removal ~ community attribute + habitat, random = 1|management). LME models were analyzed with ANOVA to obtain *p*‐values for fixed effects. Both response and explanatory variables were log10‐transformed as necessary to meet model assumptions (Crawley, 2005). We carried out the statistical analyses with r v.4.0.3 (R Development Core Team, 2019) using the libraries “nlme” (Pinheiro et al., 2017) and “FD” (Laliberté et al., 2014). Functional diversity indices were calculated using the FD function.

## 3 RESULTS

Overall, 17 species were recognized, and 4082 individuals were trapped (Table 2). The ten most abundant species (>100 individuals trapped) represented over 90% of the total catch and all three functional groups were represented in each of the two habitat categories. Overall, the ball rollers were the numerically dominant group (54% of all individuals trapped), tunnellers were moderately abundant (37%), and dwellers were relatively scarce (9%) but these proportions varied widely between habitats. Dung beetles ranged between 0.3 and 3.1 body length (cm) and between 0.67 and 5.3 (g) dry biomass per individual. Kruskal–Wallis tests showed significant statistical differences in the average species richness (H = 24.02, *df* = 1, *p* < .001) and abundance (H = 8.33, *df* = 1, *p* < .01) among open and canopied habitats and significant statistical differences in the average species richness (H = 8.51, *df* = 1, *p* < .001), and abundance among managed and natural lands (H = 3.81, *df* = 2, *p* < .001).

**TABLE 2:** Pooled estimated abundance of dung beetles active during dung degradation experiments in June–September 2019 and 2020. Total abundance for each habitat and functional group are in bold text

	Open Pasture	Canopy	Open Pasture	Canopy	Total abundance	Mean body length (cm)
	Managed	Managed	Natural	Natural		
**Tunnellers**						
*Onthophagus hecate*	183	111	85	52	**431**	**0.83**
*Onthophagus tuberculifrons*	40	184	49	77	**350**	**0.73**
*Digitonthophagus gazella*	104	56	13	0	**173**	**1.21**
*Onthophagus oklahomensis*	5	93	7	4	**109**	**0.53**
*Phanaeus vindex*	14	18	18	5	**55**	**2.9**
*Onthophagus taurus*	44	14	2	0	**60**	**0.55**
*Onthophagus pennsylvanicus*	33	92	0	3	**128**	**0.51**
*Copris minutus*	34	11	0	7	**52**	**1.7**
*Geotrupes egeriei*	42	0	28	11	**81**	**2.3**
*Euoniticellus intermedius*	41	6	17	12	**76**	**1.1**
**Total**	**540**	**585**	**219**	**171**	**1515**	
**Rollers**						
*Canthon pilularius*	43	118	238	723	**1122**	**1.9**
*Deltochilum gibbosum*	0	2	0	8	**10**	**3.1**
*Melanocanthon bispinatus*	9	3	414	451	**877**	**0.89**
*Melanocanthon punctaticollis*	3	0	34	163	**200**	**0.85**
**Total**	**55**	**123**	**686**	**1345**	**2209**	
**Dwellers**						
*Labbarus pseudolividus*	60	51	1	67	**179**	**0.45**
*Alloblackburneus campestris*	10	23	73	0	**106**	**0.34**
*Oscarinus crassulus*	13	7	23	0	**43**	**0.41**
*Irrasinus stupidus*	21	2	7	0	**30**	**0.39**
**Total**	**104**	**83**	**104**	**67**	**358**	
**Total**	**699**	**791**	**1009**	**1583**	**4082**	
Species richness	16	15	14	12	17	

### 3.1 The effect of functional diversity on dung removal

The effect of functional diversity components (FRic, FDiv, FEve) varied by management type (Table 3). Functional richness (FRic) significantly affected dung degradation in the managed (F = 2.779, *p* ≤ .001) but not the natural grassland (F = 7.410, *p* = .06); conversely, functional diversity (FDiv) had a significant impact in the natural grassland (F = 23.83, *p* = .02) but not the managed grasslands (F = 9.118, *p* = .77), and functional evenness (FEve) was not a significant factor in dung removal in either of the two management types.

**TABLE 3:** Linear mixed‐effect models to explain dung removal with community effects. Linear mixed‐effect models were analyzed with ANOVA to give *p* values for fixed effects

Community effect		
Model	*F*_1,108_	*p*
*Managed*		
Dung beetle species richness	3.358	.893
Dung beetle abundance	7.091	.**048**
Roller abundance	3.358	.982
Tunneller abundance	8.924	.**049**
Dweller abundance	0.323	.854
FRic	2.779	.**001**
FDiv	9.118	.772
FEve	0.834	.438

Natural	*F*_1,22_	*p*
Dung beetle species richness	6.821	.**081**
Dung beetle abundance	13.99	.**023**
Roller abundance	8.168	.**001**
Tunneller abundance	9.044	.**001**
Dweller abundance	1.638	.238
FRic	7.410	.**066**
FDiv	23.83	.**023**
FEve	0.892	.**052**

Note

Bold values indicate significant differences with *p* < .05.

### 3.2 Overall dung removal rate

**FIGURE 2:**
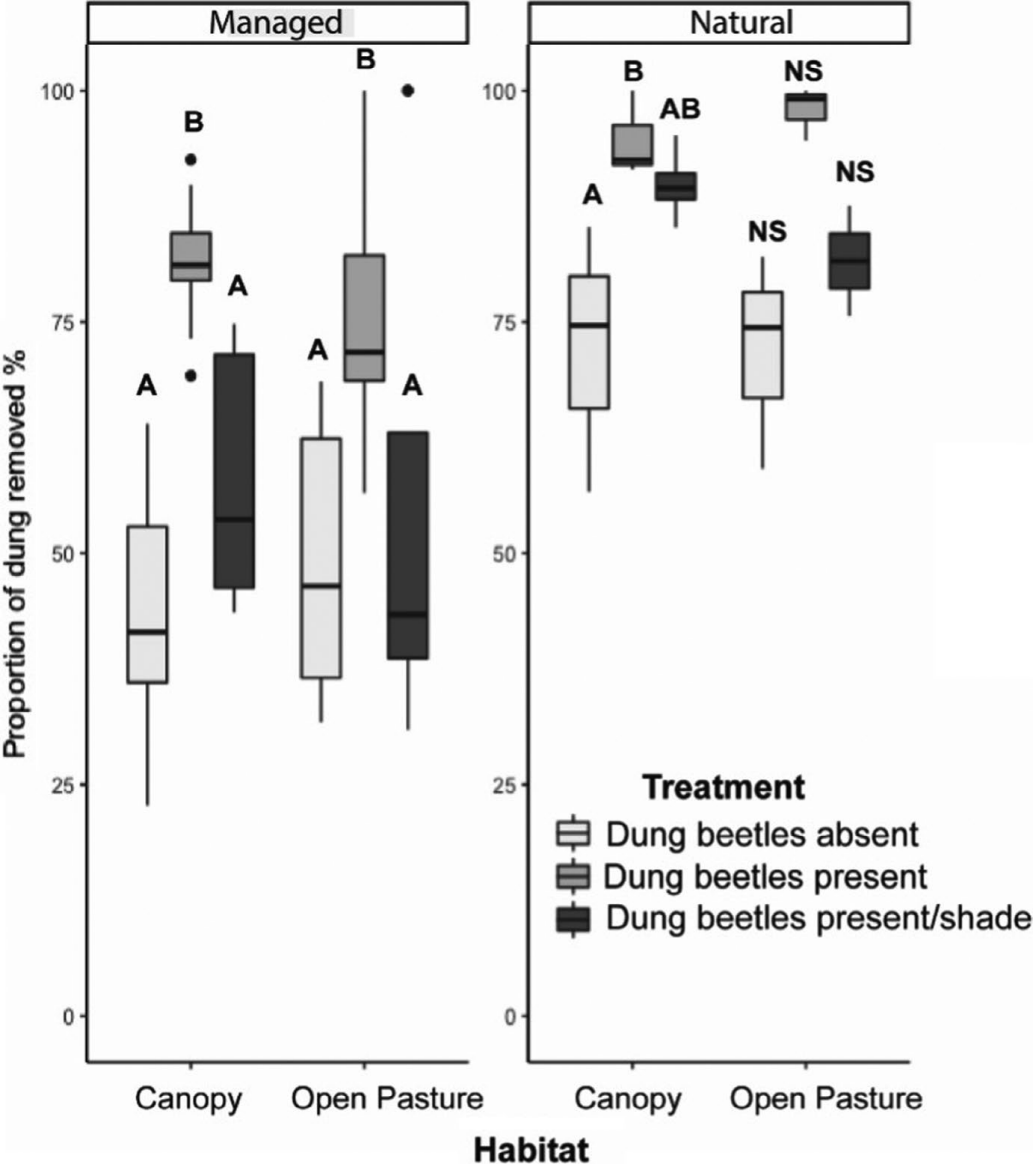
Proportion of dung removed from each habitat and management type. Different letters indicate significant differences with *p* ≤ .05, with NS being nonsignificant at *p* = .05

The contribution of dung beetles to dung removal was measured as overall mass lost at the end of the experiment. Decrease in dung weight, measured as % mass loss of the initial dung wet weight, significantly differed between treatments (H = 20.350, *df* = 3, *p* < .001) with higher loss in treatments containing the tunneller species (tunneller species only and dweller and tunneller species treatment) compared to the dung only control treatment (Figure 2). Dung removal in all sampling units was 80.6 ± 3.8% (mean 95% confidence interval). The dung was completely removed in 87 of the 120 studied sampling units (73%), and in 79% of sampling units at least half of the dung was removed. Overall, 70% of dung was removed from the soil surface after three months.

### 3.3 Differences in dung removal rate between natural and managed areas

There was a significant difference in the proportion of dung removed between the natural and managed lands (*χ* ^2^ = 31.93, *df* = 1, *p* < .001). *Post hoc* analysis revealed the median weight of dung buried by dung beetles in KPPSP was different from the dung weight buried in both ranches (Durando: z = 5.19, *p* < .001; Buck Island: z = −4.99, *p* < .001). There was no significant difference in the proportion of dung removed between the managed lands (z = 0.22, *p* = .82).

### 3.4 Effects of habitat type on dung removal rate

Overall, we found more dung was removed from soil surfaces in canopied sites compared with sites located in open pasture, irrespective of land management type (Figure 2). Canopied areas in KPPSP demonstrated a greater positive effect on dung removal (z = −82.20, *p* ≤ .001) compared to canopied sites on managed lands (z = −12.89, *p* ≤ .001).

### 3.5 Effects of dung beetle assemblage structure on dung removal rate

**FIGURE 3:**
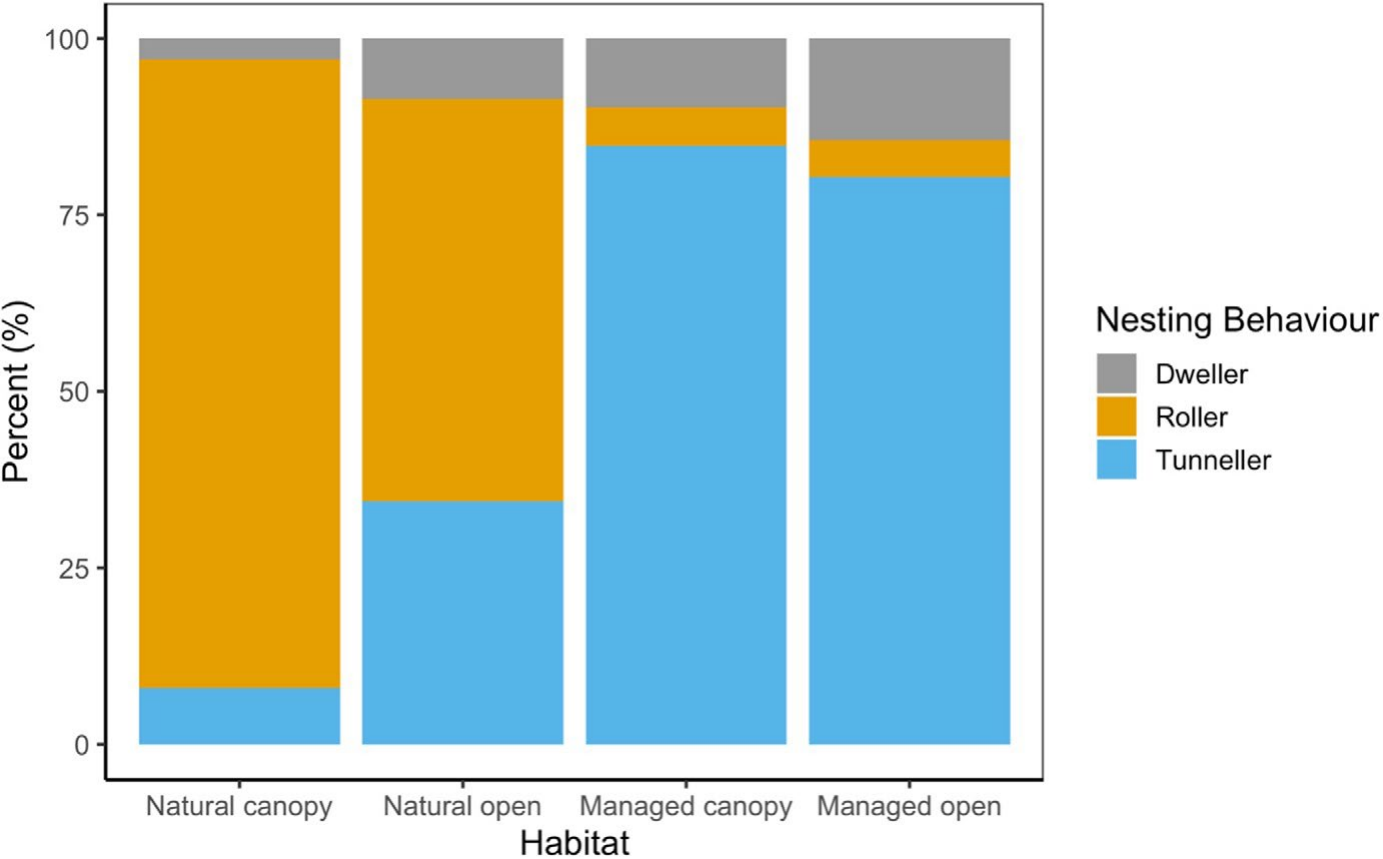
Proportion of dung beetle nesting behavior found in each habitat and management land‐use type

We found dung beetle abundance (abundance of individuals) to have a significant effect on dung removal in both the managed and natural grasslands. However, dung beetle species richness was not a significant predictor of dung removal in either land use. Small‐bodied dung beetles were demonstrated to have the largest effect on the quantity of dung removed from the soil surface after three months (z = 20.898, *p* = .001). We found significant differences between the ratio of tunnellers and rollers in managed and natural land uses with a higher proportion of rollers found in both canopied and open pasture sites in KPPSP (Figure 3).

## 4 DISCUSSION

Our results indicate that overall, dung beetle abundance and functional diversity has a substantial impact on the rate of dung turnover in subtropical pastoral lands. However, functional diversity and evenness did not have a significant positive effect on dung removal in managed grasslands demonstrating the tangible relationship between dung beetle composition and delivery of a key ecological process in landscapes affected by ecological change. While a greater proportion dung mass was lost from the control treatments in natural grasslands due to higher overall rate of evaporation, we posit that tunneller abundance was a significant factor in dung removal in both natural and managed lands while the number of tunneller species only had a significant effect on dung removal under natural land use. Tunneling dung beetles are known to be effective dung decomposers in agricultural landscapes as they are capable of removing and burying large amounts of dung in short timeframes versus rollers. Our results are consistent with those of other studies who report that dung beetle abundance, particularly the number of tunneling species plays a significant role in dung removal in subtropical grasslands (Amore et al., 2018; Correa et al., 2019; Lopez‐Collado et al., 2017). Tunneller abundance was a significant factor in dung removal in both natural and managed lands while the number of tunneller species only had a significant effect on dung removal under natural land use.

### 4.1 Landscape and dung beetle composition

Scarabaeidae dung beetles are broadly affected by landscape‐level changes that are associated with habitat destruction and alteration. Several recent studies show that areas used for cattle grazing where woodland canopy (particularly of native trees) is maintained totally or partially preserve the native diversity of dung beetles in forest ecosystems. Canopy cover has an indirect influence on dung beetles through the maintenance of soil and understory microclimatic conditions (e.g., temperature and humidity) (Davis et al., 2002; Nichols et al., 2007). Considering that forest dung beetles are characterized by a low tolerance to extreme microclimatic conditions (Costa et al., 2017; Davis & Philips, 2005; França et al., 2017), disturbances such as the removal of forested patches within agricultural mosaics which alter microclimatic factors may directly affect forest species. We suggest that the presence of canopied habitat on agropastoral lands may act as an ecological filter by modifying dung beetle assemblages and therefore may indirectly affect dung removal rates in both managed and natural subtropical grasslands where cattle graze. In contrast to another study of Florida pasture dung beetles (Conover et al., 2019), we found large tunneller species occupying mostly canopied habitat, while small tunnellers were mainly found in open grassland. Consequently, dung removal rates were up to two times higher in canopied sites compared with open grassland in both management types in our experiment. However, Conover et al. did note the effect of seasonality on the abundance of small tunnellers in canopied sites during the excessively hot and humid Florida summers with higher densities of several smaller species found during summertime compared to spring. While our study was conducted solely during the summer months, our results clearly demonstrate that the maintenance of canopied patches within agricultural mosaics likely contributes to maintaining ecosystem service provisioning in subtropical cattle pasture in this region.

Reductions in tree canopy cover, habitat fragmentation, and livestock density in agricultural landscapes have been shown to have detrimental effects on dung beetle taxonomic and functional diversity in subtropical and tropical agroecosystems (Frank et al., 2017; Gómez‐Cifuentes et al., 2017, 2019), and our results from cattle‐grazed lands in Florida follow this pattern. These landscape‐level abiotic changes impact dung beetle community assembly likely through a process of species filtering resulting in a less diverse and abundant subsample of the available decomposer community (Daniel et al., 2022). However, a recent study assessing if management practices impact dung beetle community attributes in pastures planted with exotic grasses in Brazil found that species composition, functional richness, and abundance of large rollers, and smaller tunnellers and rollers and tree density had no measurable effect on dung removal (Carvalho et al., 2021). What is unclear from these results is how extensive and distributed was the treecover given the land cover type used.

### 4.2 BEF traits and subtropical dung beetles

Broadly, the collective impact of multiple anthropogenic impacts likely affects ecosystem function and is thus of great concern. It has been repeatedly hypothesized that anthropogenic disturbance results in the extirpation of species with certain beneficial functional traits which may have outsized (relative to their population sizes or how intensively they have been studied) impacts upon ecosystem functioning (Díaz et al., 2013; Heilpern et al., 2018; Piccini et al., 2018). The relationship between biodiversity and ecosystem service provisioning is typically positive as greater species diversity leads to functionally richer assemblages which can affect multiple ecosystem services (Manning et al., 2016). The evaluation of functional diversity considers the effects that individual species traits have on ecosystems and the impact their removal may have on ecosystem stability. The differences in the amount of dung removed between natural and managed pastoral lands during our experiment demonstrate how land‐use intensity modifies BEF and provides a diminished capacity to provide valuable ecosystem functions. In particular, our results reveal that the abundance of smaller dung beetles in each study area had a significant positive effect on dung removal signaling the that overall biomass of dung beetles is more important compared to the prevalence of a few large species (Dangles et al., 2012; Tixier et al., 2015). While larger bodied dung beetle species are especially extinction prone following disturbance such as habitat fragmentation (Andresen & Laurance, 2007; Larsen et al., 2008), land‐use modification (Gardner et al., 2008), and isolation (Larsen et al., 2005), the overall loss of dung beetle biomass, including reductions in abundance which may render populations effectively functionally extinct should be of great concern to landowners and ranchers in the subtropical United States.

### 4.3 Other factors affecting dung degradation rates

Other factors may further impact declines in these beetle populations. The impact of broad‐spectrum insecticides and pesticides on populations of beneficial dung beetle species is firmly established, for example (Verdú et al., 2020), but the indirect effects of veterinary drugs on provisioning functions such as dung degradation has to date been less well explored (Tonelli et al., 2020). Due to their increased interactions with dung during manipulation on the surface, and during burial, tunnellers are known to be more susceptible to the effects of veterinary endectocides (e.g., Macrocyclic lactones) and ectocides (e.g., insect growth regulators) (Lumaret et al., 2020). Due to the substantial effect tunneling dung beetles have on driving dung degradation in US pastoral systems, it is not unreasonable to suggest that the increasing use of veterinary medicine may, along with the land‐use changes outlined in this study, have synergistic impacts which further impedes ecosystem functioning by the disruption of dung beetle lifecycles and therefore dung removal activity in US grasslands. Sustained productivity in grazing systems, including efficient recycling of nutrients and minimizing nutrient loss, depends upon soil biological processes and the interaction between grazing, nutrient mineralization, and soil biological communities. With the advent of high‐input farming and increased levels of fertilization, increased forage yields will increase the carrying capacity of pastures found in developed agricultural nations such as the United States. Without parallel increases in the dung beetle capacity to dispose of dung, or the maintenance and protection of existing dung beetle populations, large tracts of grassland will likely be fouled due to dung buildup. Slowdowns in the degradation rate of dung associated with reductions in dung beetle activity have been described in forested areas with environmental disturbance (Batilani‐Filho & Hernandez, 2017) and in agropastoral lands elsewhere (Sands et al., 2018), and it is clear that the maintenance of intact functional groups and dung relocation behavior by dung beetles in the United States provide enhanced dung removal which is essential for the continuing economic productivity of agroecosystems.

One of the more significant findings of our study indicated more dung was removed from soil surfaces in canopied sites compared with sites located in open pasture, irrespective of land management type. As livestock systems and working lands which preserve tree canopy work to maintain microclimatic conditions (Gómez‐Cifuentes et al., 2020), they provide adequate niches for dung beetle persistence. In terms of insect conservation and its complementary ecosystem functions, in subtropical pastures, we suggest that it is now imperative to keep trees dispersed within the matrixes of open pasture areas, as well as in adjacent woodlots to facilitate the exchange of dung beetles between patches within agricultural mosaics and to maintain the ecosystem services they provide. Florida is losing more than 45,000 hectares of land per year to accommodate the influx of people moving to the state, and land used for agricultural or recreational purposes is becoming increasingly threatened by development for housing. Given these pressures, the preservation of both agricultural and natural landscape mosaics is a priority in order to support healthy and robust dung beetle populations capable of providing valuable ecosystem services to agroecosystems.

## CONFLICT OF INTEREST

The authors have no conflict of interest.

## AUTHOR CONTRIBUTIONS

**Roisin Stanbrook:** Conceptualization (lead); Data curation (lead); Formal analysis (lead); Funding acquisition (supporting); Investigation (lead); Methodology (equal); Project administration (lead); Resources (supporting); Software (lead); Visualization (lead); Writing – original draft (lead); Writing – review & editing (equal). **Joshua R. King:** Conceptualization (supporting); Data curation (supporting); Formal analysis (supporting); Funding acquisition (lead); Investigation (supporting); Methodology (equal); Project administration (supporting); Resources (equal); Software (supporting); Visualization (supporting); Writing – original draft (supporting); Writing – review & editing (equal).

### OPEN RESEARCH BADGES

This article has earned an Open Data Badge for making publicly available the digitally‐shareable data necessary to reproduce the reported results. The data is available at https://doi.org/10.5061/dryad.brv15dvbk.

## Supporting information

Figure S1

## Data Availability

The data presented in this study are openly available in Dryad at https://doi.org/10.5061/dryad.brv15dvbk
