# Supplementary figures and images for: Dung beetle community composition affects dung turnover in subtropical US grasslands
Source: Ecol Evol. 2022 Feb 22;12(2):e8660. doi: 10.1002/ece3.8660 (PMC8861836; doi:10.1002/ece3.8660)

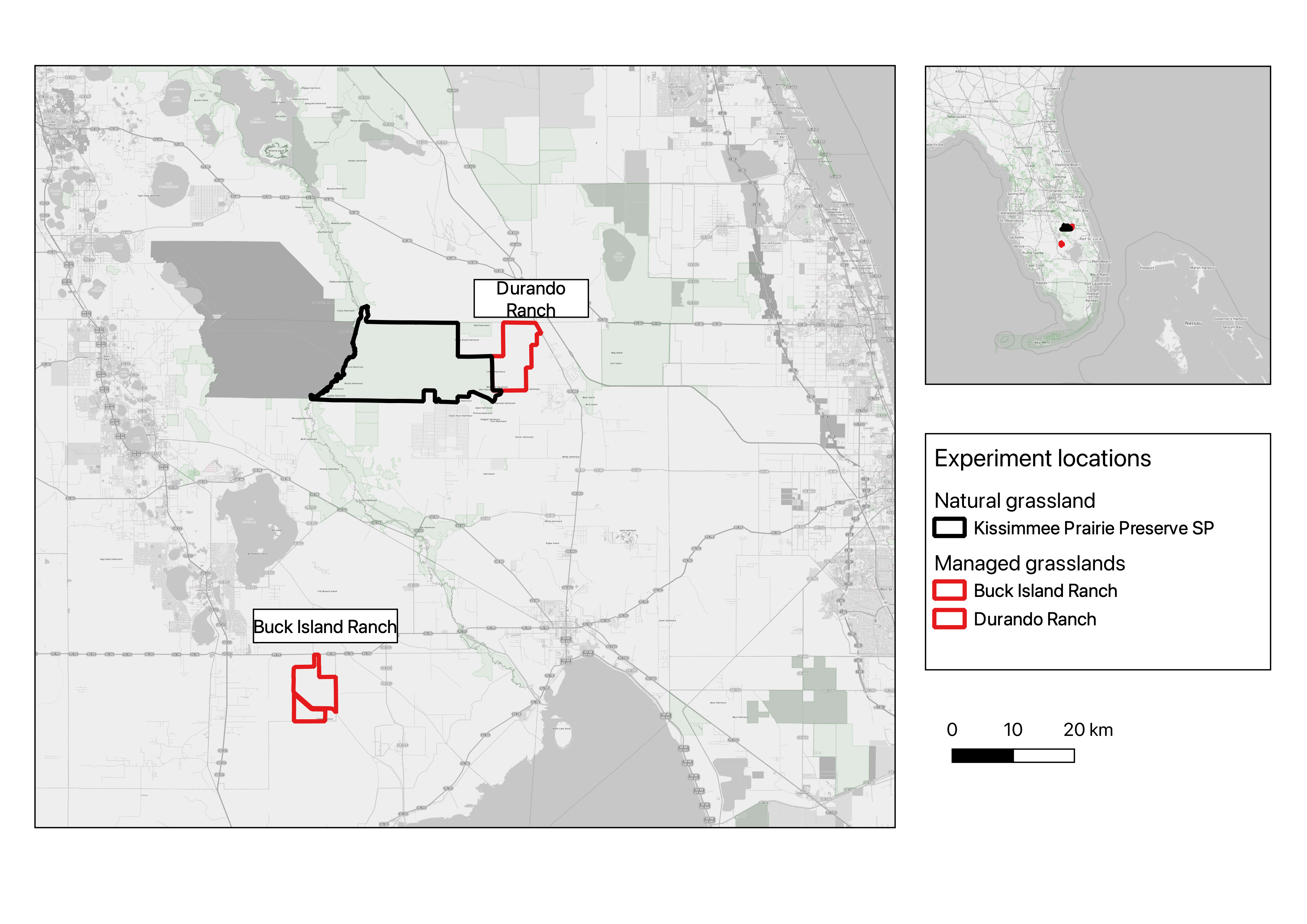

Supplement: Supplementary file 1 — Figure S1 [file ECE3-12-e8660-s001.tif]
